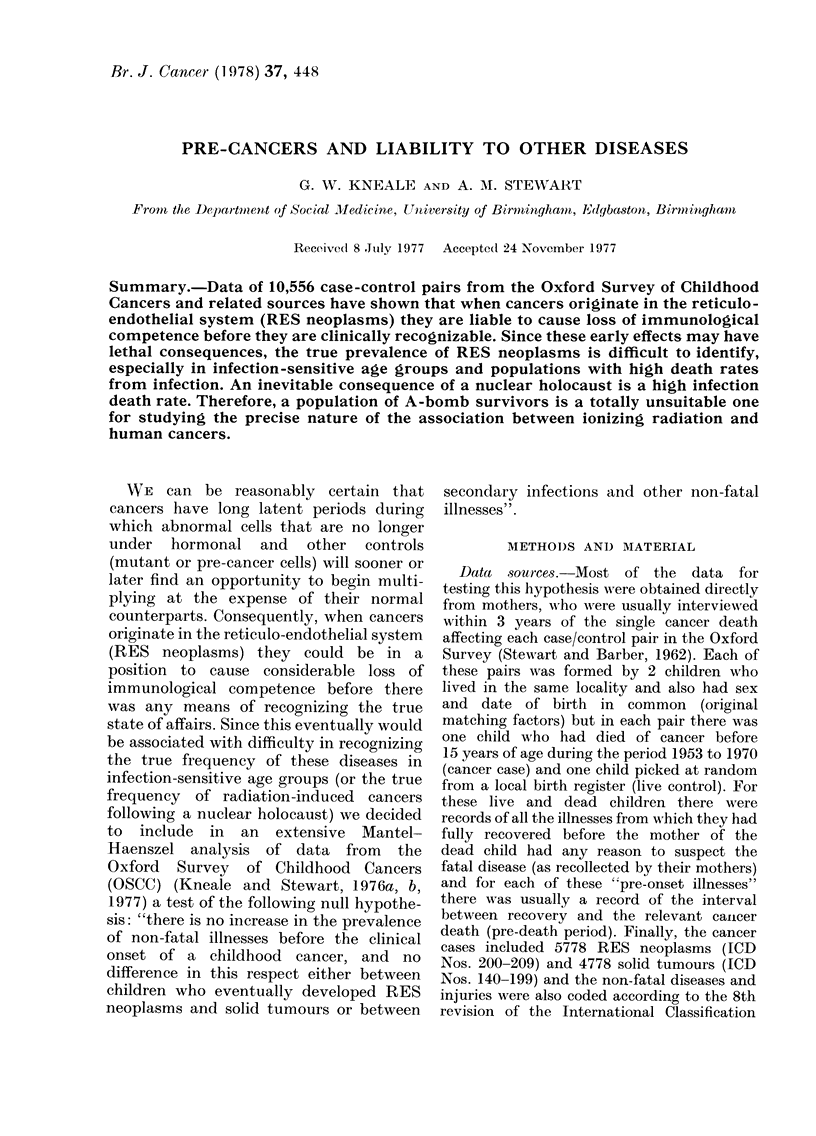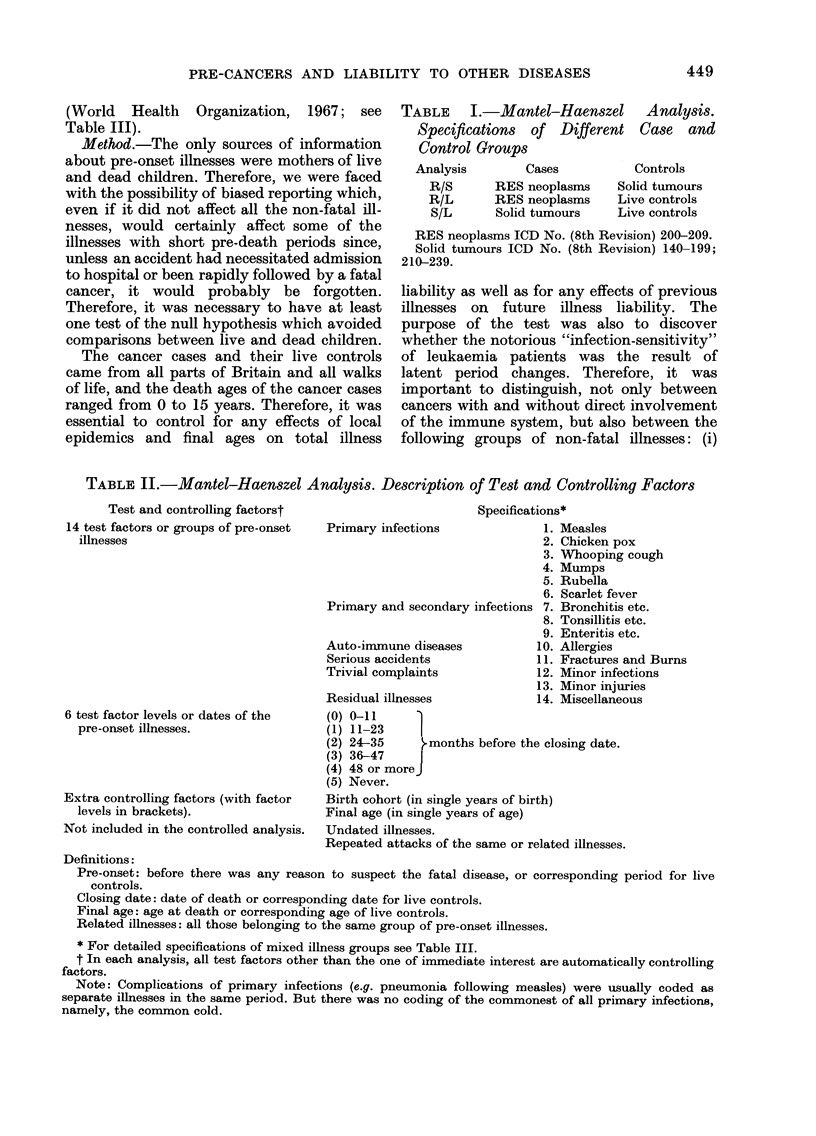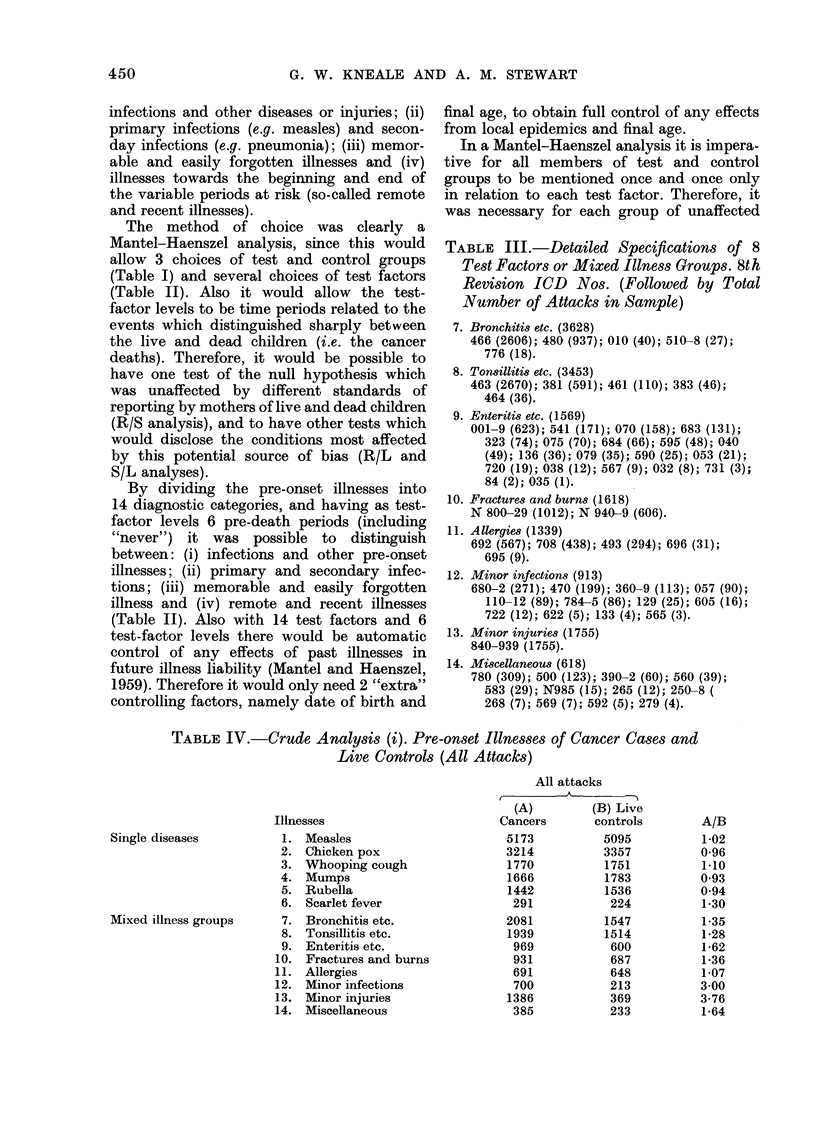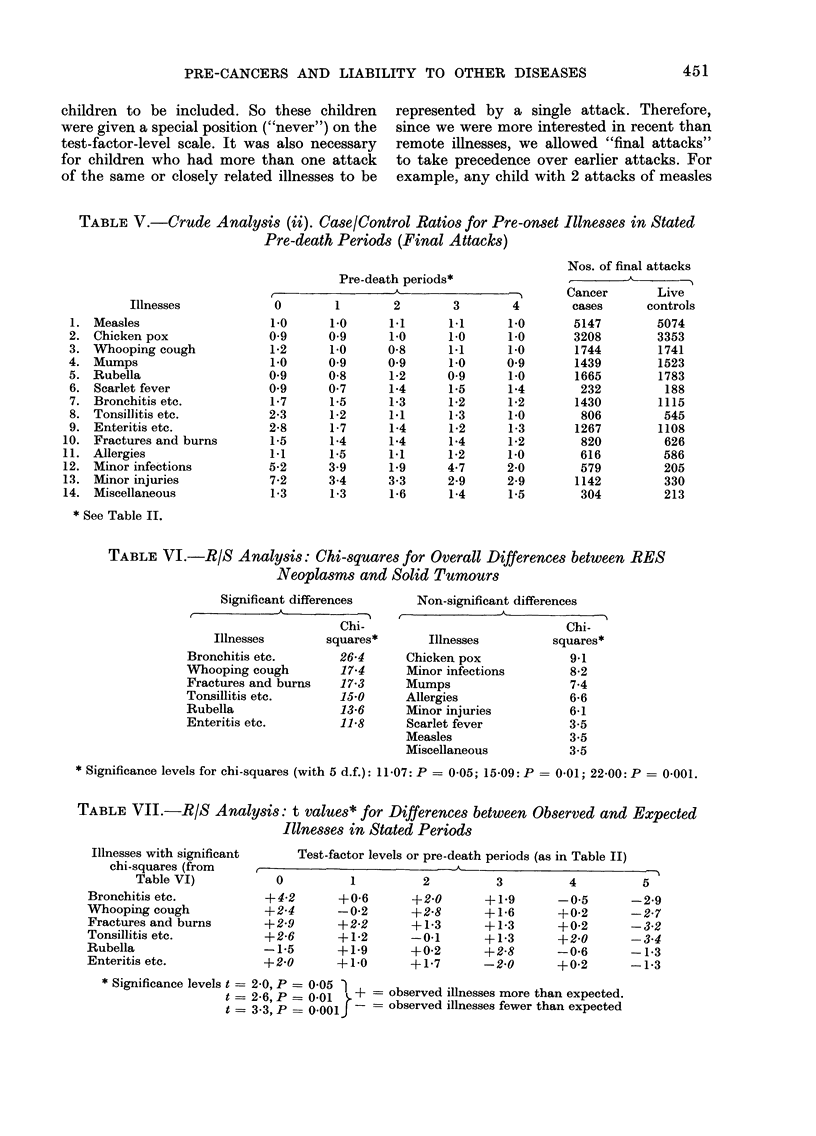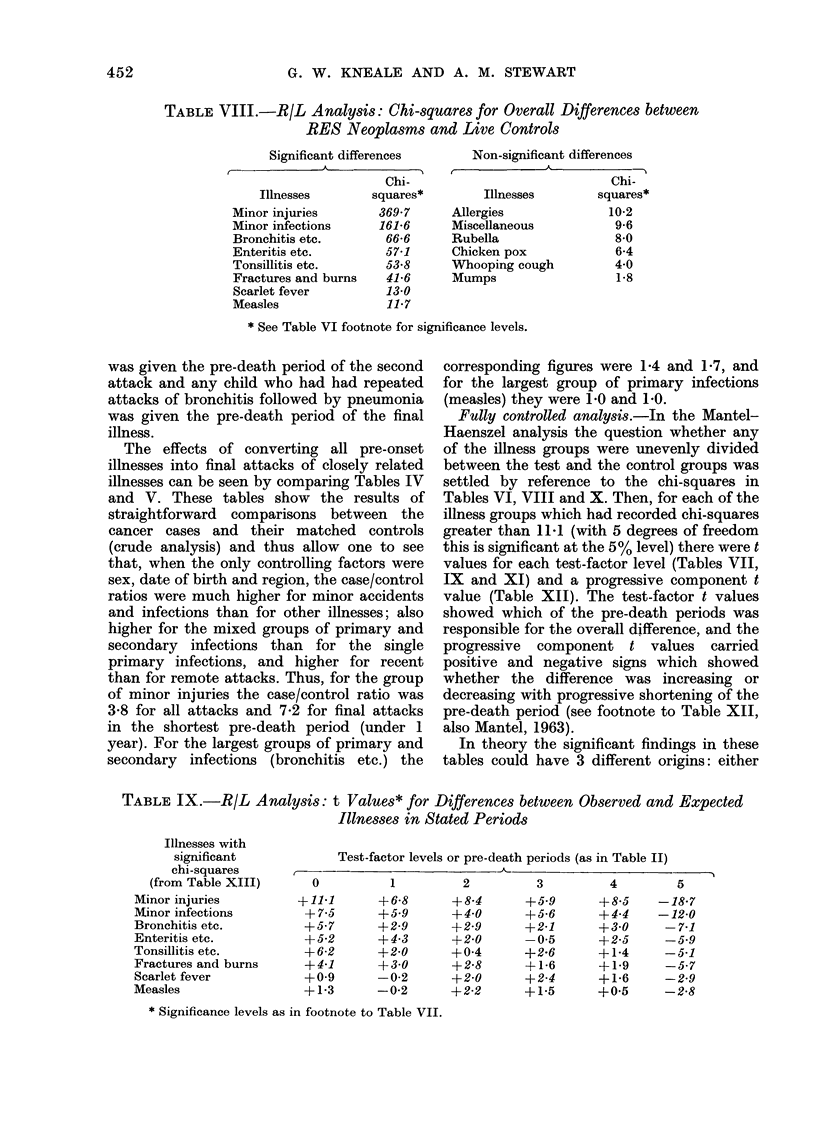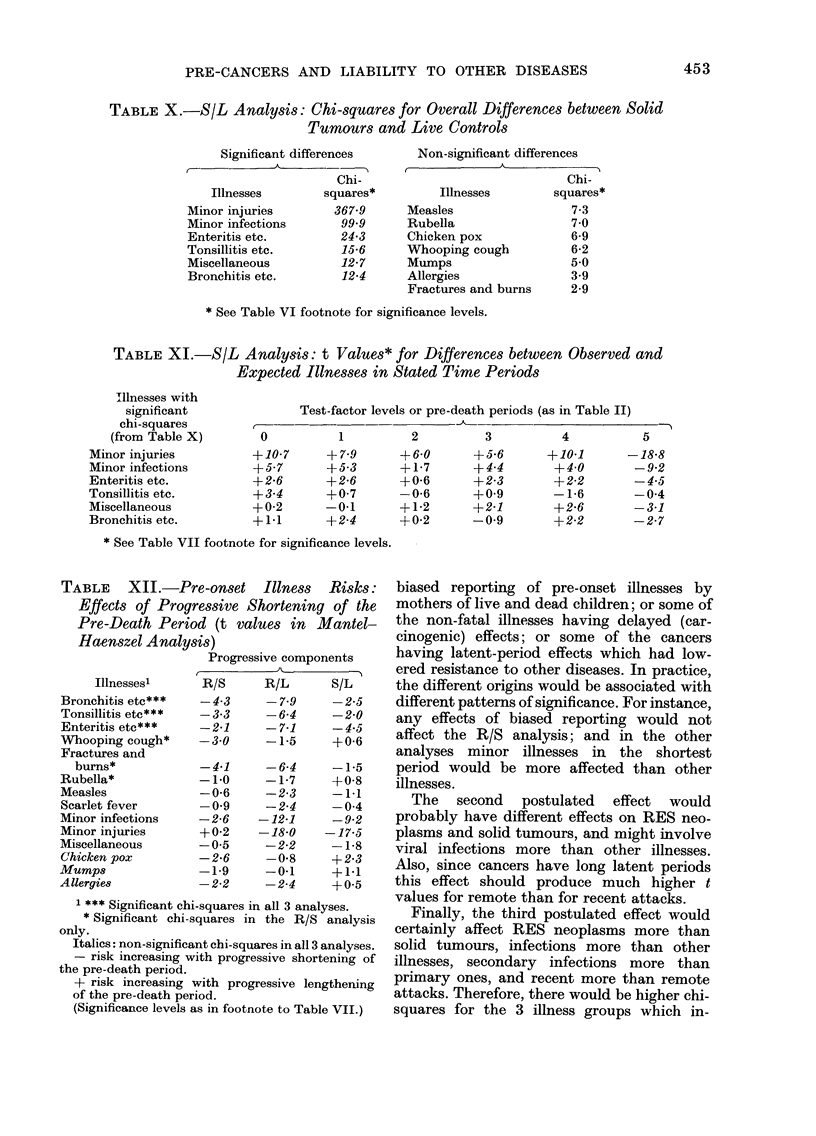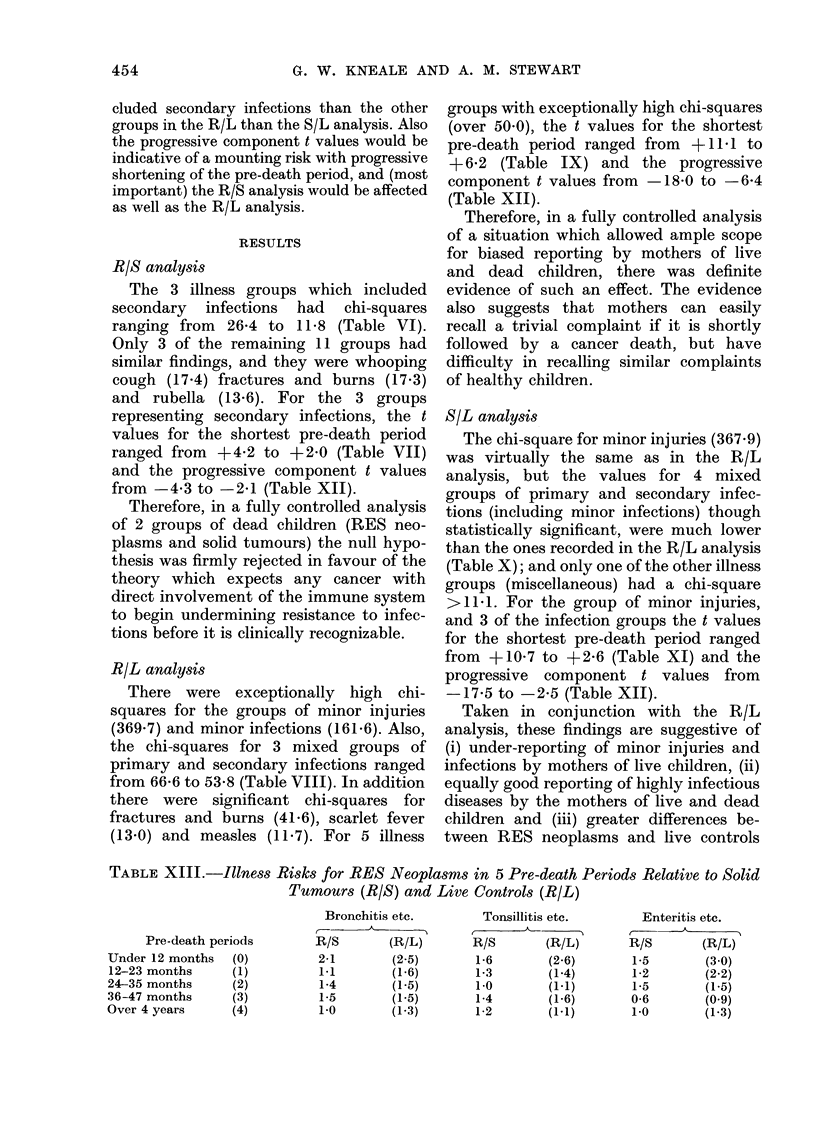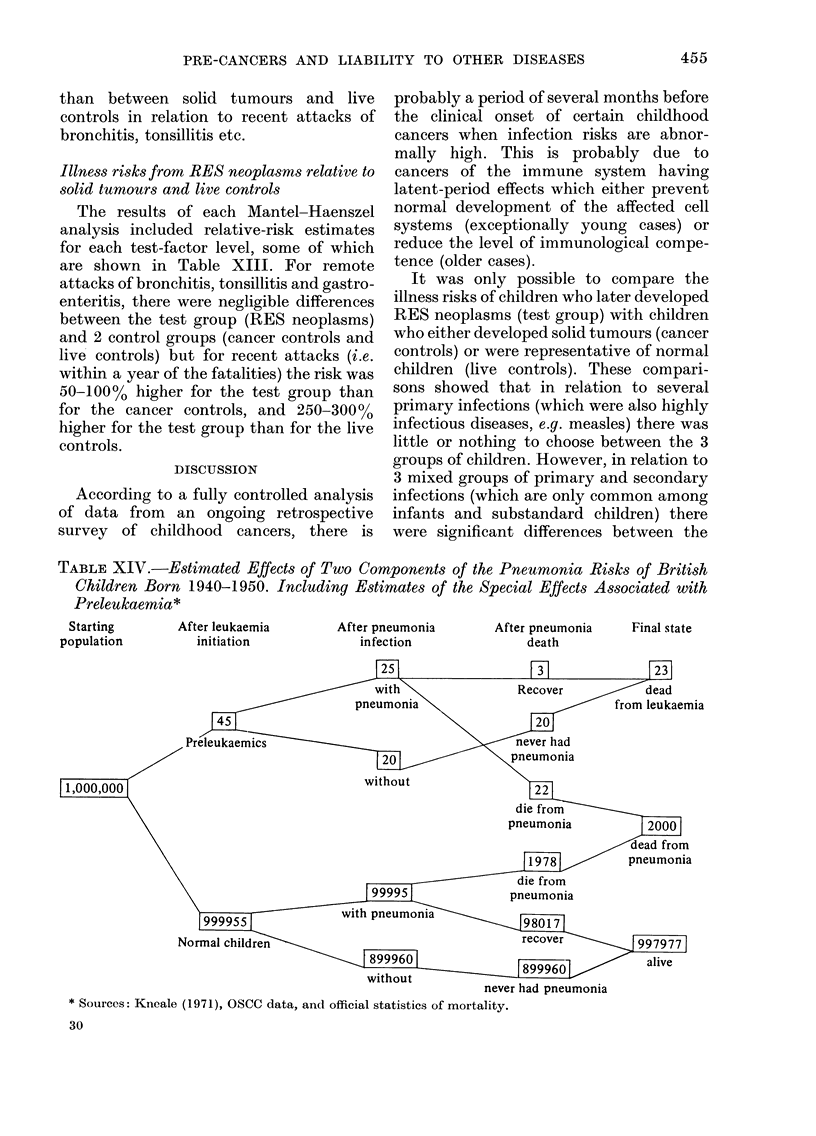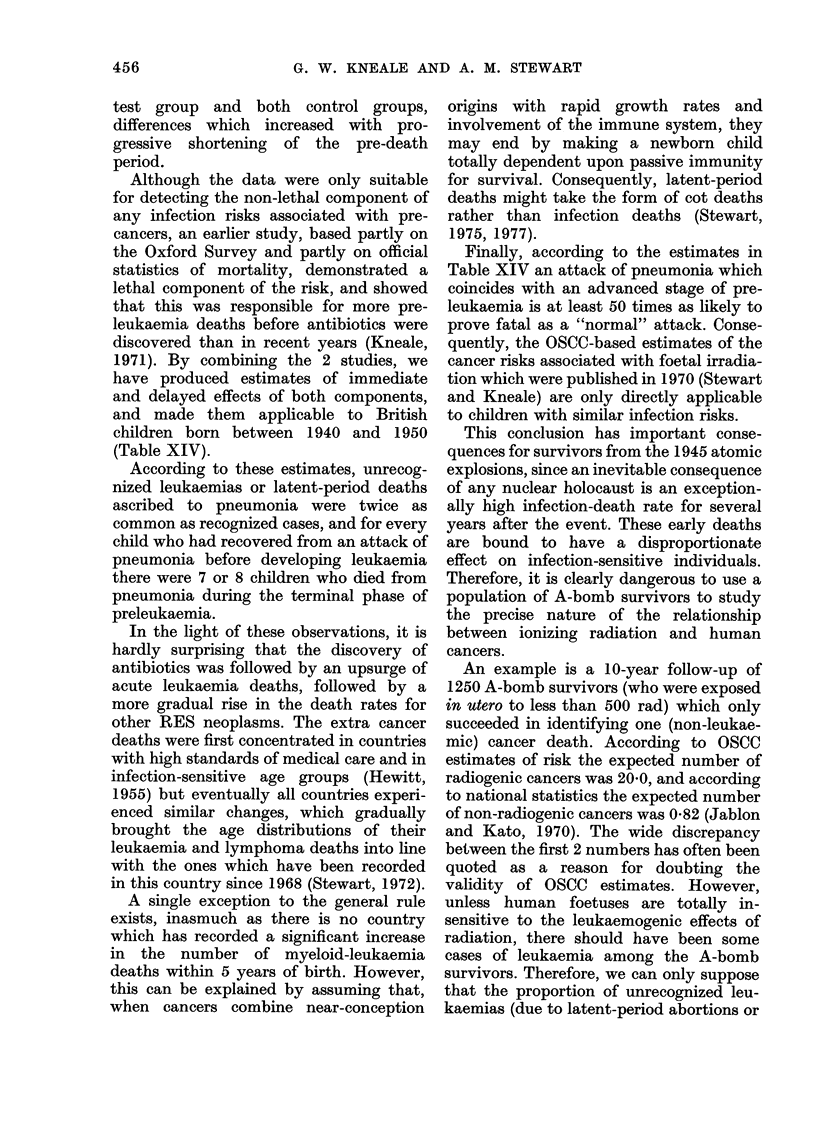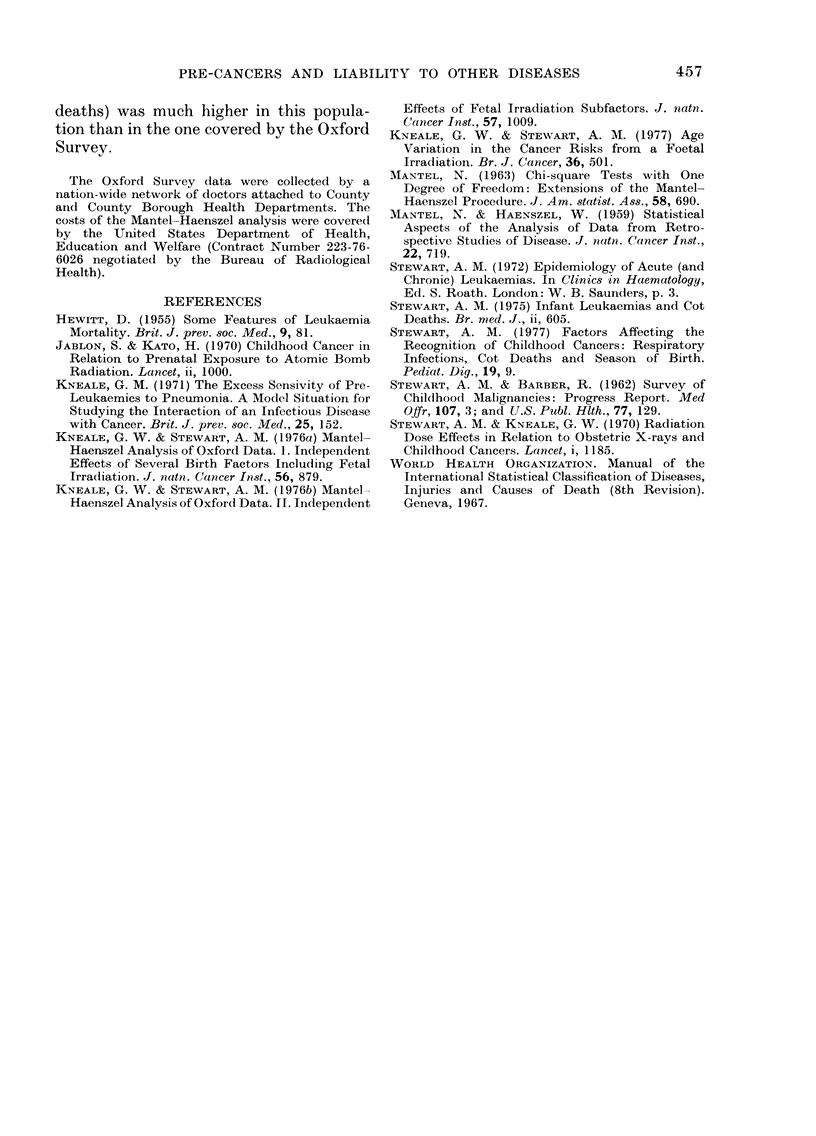# Pre-cancers and liability to other diseases.

**DOI:** 10.1038/bjc.1978.65

**Published:** 1978-03

**Authors:** G. W. Kneale, A. M. Stewart

## Abstract

Data of 10,556 case-control pairs from the Oxford Survey of Childhood Cancers and related sources have shown that when cancers originate in the reticuloendothelial system (RES neoplasms) they are liable to cause loss of immunological competence before they are clinically recognizable. Since these early effects may have lethal consequences, the true prevalence of RES neoplasms is difficult to identify, especially in infection-sensitive age groups and populations with high death rates from infection. An inevitable consequence of a nuclear holocaust is a high infection death rate. Therefore, a population of A-bomb survivors is a totally unsuitable one for studying the precise nature of the association between ionizing radiation and human cancers.


*Br. J. Cancer* (1978) **37**, 448

### PRE-CANCERS AND LIABILITY TO OTHER DISEASES

G. W. KNEALE AND A. M. STEWART

*From the Department of Social Medicine, University of Birmingham, Edgbaston, Birmingham*

Received 8 July 1977 Accepted 24 November 1977

**Summary.—Data of 10,556 case-control pairs from the Oxford Survey of Childhood Cancers and related sources have shown that when cancers originate in the reticulo-endothelial system (RES neoplasms) they are liable to cause loss of immunological competence before they are clinically recognizable. Since these early effects may have lethal consequences, the true prevalence of RES neoplasms is difficult to identify, especially in infection-sensitive age groups and populations with high death rates from infection. An inevitable consequence of a nuclear holocaust is a high infection death rate. Therefore, a population of A-bomb survivors is a totally unsuitable one for studying the precise nature of the association between ionizing radiation and human cancers.**

We can be reasonably certain that cancers have long latent periods during which abnormal cells that are no longer under hormonal and other controls (mutant or pre-cancer cells) will sooner or later find an opportunity to begin multiplying at the expense of their normal counterparts. Consequently, when cancers originate in the reticulo-endothelial system (RES neoplasms) they could be in a position to cause considerable loss of immunological competence before there was any means of recognizing the true state of affairs. Since this eventually would be associated with difficulty in recognizing the true frequency of these diseases in infection-sensitive age groups (or the true frequency of radiation-induced cancers following a nuclear holocaust) we decided to include in an extensive Mantel–Haenszel analysis of data from the Oxford Survey of Childhood Cancers (OSCC) (Kneale and Stewart, 1976*a*, *b*, 1977) a test of the following null hypothesis: "there is no increase in the prevalence of non-fatal illnesses before the clinical onset of a childhood cancer, and no difference in this respect either between children who eventually developed RES neoplasms and solid tumours or between secondary infections and other non-fatal illnesses".

### METHODS AND MATERIAL

*Data sources.*—Most of the data for testing this hypothesis were obtained directly from mothers, who were usually interviewed within 3 years of the single cancer death affecting each case/control pair in the Oxford Survey (Stewart and Barber, 1962). Each of these pairs was formed by 2 children who lived in the same locality and also had sex and date of birth in common (original matching factors) but in each pair there was one child who had died of cancer before 15 years of age during the period 1953 to 1970 (cancer case) and one child picked at random from a local birth register (live control). For these live and dead children there were records of all the illnesses from which they had fully recovered before the mother of the dead child had any reason to suspect the fatal disease (as recollected by their mothers) and for each of these "pre-onset illnesses" there was usually a record of the interval between recovery and the relevant cancer death (pre-death period). Finally, the cancer cases included 5778 RES neoplasms (ICD Nos. 200–209) and 4778 solid tumours (ICD Nos. 140–199) and the non-fatal diseases and injuries were also coded according to the 8th revision of the International Classification

(World Health Organization, 1967; see Table III).

*Method.*—The only sources of information about pre-onset illnesses were mothers of live and dead children. Therefore, we were faced with the possibility of biased reporting which, even if it did not affect all the non-fatal illnesses, would certainly affect some of the illnesses with short pre-death periods since, unless an accident had necessitated admission to hospital or been rapidly followed by a fatal cancer, it would probably be forgotten. Therefore, it was necessary to have at least one test of the null hypothesis which avoided comparisons between live and dead children.

The cancer cases and their live controls came from all parts of Britain and all walks of life, and the death ages of the cancer cases ranged from 0 to 15 years. Therefore, it was essential to control for any effects of local epidemics and final ages on total illness liability as well as for any effects of previous illnesses on future illness liability. The purpose of the test was also to discover whether the notorious "infection-sensitivity" of leukaemia patients was the result of latent period changes. Therefore, it was important to distinguish, not only between cancers with and without direct involvement of the immune system, but also between the following groups of non-fatal illnesses: (i)

TABLE I.—*Mantel–Haenszel Analysis. Specifications of Different Case and Control Groups*

| Analysis | Cases | Controls |
|---|---|---|
| R/S | RES neoplasms | Solid tumours |
| R/L | RES neoplasms | Live controls |
| S/L | Solid tumours | Live controls |

RES neoplasms ICD No. (8th Revision) 200–209.
Solid tumours ICD No. (8th Revision) 140–199; 210–239.

TABLE II.—*Mantel–Haenszel Analysis. Description of Test and Controlling Factors*

| Test and controlling factors† | Specifications* | |
|---|---|---|
| 14 test factors or groups of pre-onset illnesses | Primary infections | 1. Measles |
| | | 2. Chicken pox |
| | | 3. Whooping cough |
| | | 4. Mumps |
| | | 5. Rubella |
| | | 6. Scarlet fever |
| | Primary and secondary infections | 7. Bronchitis etc. |
| | | 8. Tonsillitis etc. |
| | | 9. Enteritis etc. |
| | Auto-immune diseases | 10. Allergies |
| | Serious accidents | 11. Fractures and Burns |
| | Trivial complaints | 12. Minor infections |
| | | 13. Minor injuries |
| | Residual illnesses | 14. Miscellaneous |
| 6 test factor levels or dates of the pre-onset illnesses. | (0) 0–11 | months before the closing date. |
| | (1) 11–23 | months before the closing date. |
| | (2) 24–35 | months before the closing date. |
| | (3) 36–47 | months before the closing date. |
| | (4) 48 or more | months before the closing date. |
| | (5) Never. | |
| Extra controlling factors (with factor levels in brackets). | Birth cohort (in single years of birth) | |
| | Final age (in single years of age) | |
| Not included in the controlled analysis. | Undated illnesses. | |
| | Repeated attacks of the same or related illnesses. | |

Definitions:
Pre-onset: before there was any reason to suspect the fatal disease, or corresponding period for live controls.
Closing date: date of death or corresponding date for live controls.
Final age: age at death or corresponding age of live controls.
Related illnesses: all those belonging to the same group of pre-onset illnesses.

* For detailed specifications of mixed illness groups see Table III.
† In each analysis, all test factors other than the one of immediate interest are automatically controlling factors.
Note: Complications of primary infections (*e.g.* pneumonia following measles) were usually coded as separate illnesses in the same period. But there was no coding of the commonest of all primary infections, namely, the common cold.

infections and other diseases or injuries; (ii) primary infections (*e.g.* measles) and seconday infections (*e.g.* pneumonia); (iii) memorable and easily forgotten illnesses and (iv) illnesses towards the beginning and end of the variable periods at risk (so-called remote and recent illnesses).

The method of choice was clearly a Mantel–Haenszel analysis, since this would allow 3 choices of test and control groups (Table I) and several choices of test factors (Table II). Also it would allow the test-factor levels to be time periods related to the events which distinguished sharply between the live and dead children (*i.e.* the cancer deaths). Therefore, it would be possible to have one test of the null hypothesis which was unaffected by different standards of reporting by mothers of live and dead children (R/S analysis), and to have other tests which would disclose the conditions most affected by this potential source of bias (R/L and S/L analyses).

By dividing the pre-onset illnesses into 14 diagnostic categories, and having as test-factor levels 6 pre-death periods (including "never") it was possible to distinguish between: (i) infections and other pre-onset illnesses; (ii) primary and secondary infections; (iii) memorable and easily forgotten illness and (iv) remote and recent illnesses (Table II). Also with 14 test factors and 6 test-factor levels there would be automatic control of any effects of past illnesses in future illness liability (Mantel and Haenszel, 1959). Therefore it would only need 2 "extra" controlling factors, namely date of birth and final age, to obtain full control of any effects from local epidemics and final age.

In a Mantel–Haenszel analysis it is imperative for all members of test and control groups to be mentioned once and once only in relation to each test factor. Therefore, it was necessary for each group of unaffected

TABLE III.—*Detailed Specifications of* 8 *Test Factors or Mixed Illness Groups.* 8*th Revision ICD Nos.* (*Followed by Total Number of Attacks in Sample*)

7. *Bronchitis etc.* (3628)
   466 (2606); 480 (937); 010 (40); 510–8 (27); 776 (18).
8. *Tonsillitis etc.* (3453)
   463 (2670); 381 (591); 461 (110); 383 (46); 464 (36).
9. *Enteritis etc.* (1569)
   001–9 (623); 541 (171); 070 (158); 683 (131); 323 (74); 075 (70); 684 (66); 595 (48); 040 (49); 136 (36); 079 (35); 590 (25); 053 (21); 720 (19); 038 (12); 567 (9); 032 (8); 731 (3); 84 (2); 035 (1).
10. *Fractures and burns* (1618)
    N 800–29 (1012); N 940–9 (606).
11. *Allergies* (1339)
    692 (567); 708 (438); 493 (294); 696 (31); 695 (9).
12. *Minor infections* (913)
    680–2 (271); 470 (199); 360–9 (113); 057 (90); 110–12 (89); 784–5 (86); 129 (25); 605 (16); 722 (12); 622 (5); 133 (4); 565 (3).
13. *Minor injuries* (1755)
    840–939 (1755).
14. *Miscellaneous* (618)
    780 (309); 500 (123); 390–2 (60); 560 (39); 583 (29); N985 (15); 265 (12); 250–8 ( 268 (7); 569 (7); 592 (5); 279 (4).

TABLE IV.—*Crude Analysis* (*i*). *Pre-onset Illnesses of Cancer Cases and Live Controls* (*All Attacks*)

| | Illnesses | All attacks (A) Cancers | All attacks (B) Live controls | A/B |
|---|---|---|---|---|
| Single diseases | 1. Measles | 5173 | 5095 | 1·02 |
| | 2. Chicken pox | 3214 | 3357 | 0·96 |
| | 3. Whooping cough | 1770 | 1751 | 1·10 |
| | 4. Mumps | 1666 | 1783 | 0·93 |
| | 5. Rubella | 1442 | 1536 | 0·94 |
| | 6. Scarlet fever | 291 | 224 | 1·30 |
| Mixed illness groups | 7. Bronchitis etc. | 2081 | 1547 | 1·35 |
| | 8. Tonsillitis etc. | 1939 | 1514 | 1·28 |
| | 9. Enteritis etc. | 969 | 600 | 1·62 |
| | 10. Fractures and burns | 931 | 687 | 1·36 |
| | 11. Allergies | 691 | 648 | 1·07 |
| | 12. Minor infections | 700 | 213 | 3·00 |
| | 13. Minor injuries | 1386 | 369 | 3·76 |
| | 14. Miscellaneous | 385 | 233 | 1·64 |

children to be included. So these children were given a special position ("never") on the test-factor-level scale. It was also necessary for children who had more than one attack of the same or closely related illnesses to be represented by a single attack. Therefore, since we were more interested in recent than remote illnesses, we allowed "final attacks" to take precedence over earlier attacks. For example, any child with 2 attacks of measles

TABLE V.—*Crude Analysis (ii). Case/Control Ratios for Pre-onset Illnesses in Stated Pre-death Periods (Final Attacks)*

| | Illnesses | Pre-death periods* 0 | 1 | 2 | 3 | 4 | Nos. of final attacks: Cancer cases | Live controls |
|---|---|---|---|---|---|---|---|---|
| 1. | Measles | 1·0 | 1·0 | 1·1 | 1·1 | 1·0 | 5147 | 5074 |
| 2. | Chicken pox | 0·9 | 0·9 | 1·0 | 1·0 | 1·0 | 3208 | 3353 |
| 3. | Whooping cough | 1·2 | 1·0 | 0·8 | 1·1 | 1·0 | 1744 | 1741 |
| 4. | Mumps | 1·0 | 0·9 | 0·9 | 1·0 | 0·9 | 1439 | 1523 |
| 5. | Rubella | 0·9 | 0·8 | 1·2 | 0·9 | 1·0 | 1665 | 1783 |
| 6. | Scarlet fever | 0·9 | 0·7 | 1·4 | 1·5 | 1·4 | 232 | 188 |
| 7. | Bronchitis etc. | 1·7 | 1·5 | 1·3 | 1·2 | 1·2 | 1430 | 1115 |
| 8. | Tonsillitis etc. | 2·3 | 1·2 | 1·1 | 1·3 | 1·0 | 806 | 545 |
| 9. | Enteritis etc. | 2·8 | 1·7 | 1·4 | 1·2 | 1·3 | 1267 | 1108 |
| 10. | Fractures and burns | 1·5 | 1·4 | 1·4 | 1·4 | 1·2 | 820 | 626 |
| 11. | Allergies | 1·1 | 1·5 | 1·1 | 1·2 | 1·0 | 616 | 586 |
| 12. | Minor infections | 5·2 | 3·9 | 1·9 | 4·7 | 2·0 | 579 | 205 |
| 13. | Minor injuries | 7·2 | 3·4 | 3·3 | 2·9 | 2·9 | 1142 | 330 |
| 14. | Miscellaneous | 1·3 | 1·3 | 1·6 | 1·4 | 1·5 | 304 | 213 |

* See Table II.

TABLE VI.—*R/S Analysis: Chi-squares for Overall Differences between RES Neoplasms and Solid Tumours*

| Significant differences: Illnesses | Chi-squares* | Non-significant differences: Illnesses | Chi-squares* |
|---|---|---|---|
| Bronchitis etc. | *26·4* | Chicken pox | 9·1 |
| Whooping cough | *17·4* | Minor infections | 8·2 |
| Fractures and burns | *17·3* | Mumps | 7·4 |
| Tonsillitis etc. | *15·0* | Allergies | 6·6 |
| Rubella | *13·6* | Minor injuries | 6·1 |
| Enteritis etc. | *11·8* | Scarlet fever | 3·5 |
| | | Measles | 3·5 |
| | | Miscellaneous | 3·5 |

* Significance levels for chi-squares (with 5 d.f.): 11·07: $P = 0{\cdot}05$; 15·09: $P = 0{\cdot}01$; 22·00: $P = 0{\cdot}001$.

TABLE VII.—*R/S Analysis:* t *values* for Differences between Observed and Expected Illnesses in Stated Periods*

| Illnesses with significant chi-squares (from Table VI) | Test-factor levels or pre-death periods (as in Table II) 0 | 1 | 2 | 3 | 4 | 5 |
|---|---|---|---|---|---|---|
| Bronchitis etc. | *+4·2* | +0·6 | *+2·0* | +1·9 | −0·5 | −2·9 |
| Whooping cough | *+2·4* | −0·2 | *+2·8* | +1·6 | +0·2 | *−2·7* |
| Fractures and burns | *+2·9* | *+2·2* | +1·3 | +1·3 | +0·2 | *−3·2* |
| Tonsillitis etc. | *+2·6* | +1·2 | −0·1 | +1·3 | *+2·0* | *−3·4* |
| Rubella | −1·5 | +1·9 | +0·2 | *+2·8* | −0·6 | −1·3 |
| Enteritis etc. | *+2·0* | +1·0 | +1·7 | *−2·0* | +0·2 | −1·3 |

* Significance levels $t = 2{\cdot}0, P = 0{\cdot}05$; $t = 2{\cdot}6, P = 0{\cdot}01$; $t = 3{\cdot}3, P = 0{\cdot}001$.
+ = observed illnesses more than expected.
− = observed illnesses fewer than expected

TABLE VIII.—*R/L Analysis: Chi-squares for Overall Differences between RES Neoplasms and Live Controls*

| Significant differences: Illnesses | Chi-squares* | Non-significant differences: Illnesses | Chi-squares* |
|---|---|---|---|
| Minor injuries | *369·7* | Allergies | 10·2 |
| Minor infections | *161·6* | Miscellaneous | 9·6 |
| Bronchitis etc. | *66·6* | Rubella | 8·0 |
| Enteritis etc. | *57·1* | Chicken pox | 6·4 |
| Tonsillitis etc. | *53·8* | Whooping cough | 4·0 |
| Fractures and burns | *41·6* | Mumps | 1·8 |
| Scarlet fever | *13·0* | | |
| Measles | *11·7* | | |

* See Table VI footnote for significance levels.

was given the pre-death period of the second attack and any child who had had repeated attacks of bronchitis followed by pneumonia was given the pre-death period of the final illness.

The effects of converting all pre-onset illnesses into final attacks of closely related illnesses can be seen by comparing Tables IV and V. These tables show the results of straightforward comparisons between the cancer cases and their matched controls (crude analysis) and thus allow one to see that, when the only controlling factors were sex, date of birth and region, the case/control ratios were much higher for minor accidents and infections than for other illnesses; also higher for the mixed groups of primary and secondary infections than for the single primary infections, and higher for recent than for remote attacks. Thus, for the group of minor injuries the case/control ratio was 3·8 for all attacks and 7·2 for final attacks in the shortest pre-death period (under 1 year). For the largest groups of primary and secondary infections (bronchitis etc.) the corresponding figures were 1·4 and 1·7, and for the largest group of primary infections (measles) they were 1·0 and 1·0.

*Fully controlled analysis.*—In the Mantel–Haenszel analysis the question whether any of the illness groups were unevenly divided between the test and the control groups was settled by reference to the chi-squares in Tables VI, VIII and X. Then, for each of the illness groups which had recorded chi-squares greater than 11·1 (with 5 degrees of freedom this is significant at the 5% level) there were *t* values for each test-factor level (Tables VII, IX and XI) and a progressive component *t* value (Table XII). The test-factor *t* values showed which of the pre-death periods was responsible for the overall difference, and the progressive component *t* values carried positive and negative signs which showed whether the difference was increasing or decreasing with progressive shortening of the pre-death period (see footnote to Table XII, also Mantel, 1963).

In theory the significant findings in these tables could have 3 different origins: either

TABLE IX.—*R/L Analysis:* t *Values* for Differences between Observed and Expected Illnesses in Stated Periods*

| Illnesses with significant chi-squares (from Table XIII) | Test-factor levels or pre-death periods (as in Table II): 0 | 1 | 2 | 3 | 4 | 5 |
|---|---|---|---|---|---|---|
| Minor injuries | *+11·1* | *+6·8* | *+8·4* | *+5·9* | *+8·5* | *−18·7* |
| Minor infections | *+7·5* | *+5·9* | *+4·0* | *+5·6* | *+4·4* | *−12·0* |
| Bronchitis etc. | *+5·7* | *+2·9* | *+2·9* | *+2·1* | *+3·0* | *−7·1* |
| Enteritis etc. | *+5·2* | *+4·3* | *+2·0* | −0·5 | *+2·5* | *−5·9* |
| Tonsillitis etc. | *+6·2* | *+2·0* | +0·4 | *+2·6* | +1·4 | *−5·1* |
| Fractures and burns | *+4·1* | *+3·0* | *+2·8* | +1·6 | +1·9 | *−5·7* |
| Scarlet fever | +0·9 | −0·2 | *+2·0* | *+2·4* | +1·6 | *−2·9* |
| Measles | +1·3 | −0·2 | *+2·2* | +1·5 | +0·5 | *−2·8* |

* Significance levels as in footnote to Table VII.

TABLE X.—*S/L Analysis: Chi-squares for Overall Differences between Solid Tumours and Live Controls*

| Significant differences: Illnesses | Chi-squares* | Non-significant differences: Illnesses | Chi-squares* |
|---|---|---|---|
| Minor injuries | *367·9* | Measles | 7·3 |
| Minor infections | *99·9* | Rubella | 7·0 |
| Enteritis etc. | *24·3* | Chicken pox | 6·9 |
| Tonsillitis etc. | *15·6* | Whooping cough | 6·2 |
| Miscellaneous | *12·7* | Mumps | 5·0 |
| Bronchitis etc. | *12·4* | Allergies | 3·9 |
| | | Fractures and burns | 2·9 |

* See Table VI footnote for significance levels.

TABLE XI.—*S/L Analysis:* t *Values* for Differences between Observed and Expected Illnesses in Stated Time Periods*

| Illnesses with significant chi-squares (from Table X) | Test-factor levels or pre-death periods (as in Table II): 0 | 1 | 2 | 3 | 4 | 5 |
|---|---|---|---|---|---|---|
| Minor injuries | +*10·7* | +*7·9* | +*6·0* | +*5·6* | +*10·1* | −*18·8* |
| Minor infections | +*5·7* | +*5·3* | +1·7 | +*4·4* | +*4·0* | −*9·2* |
| Enteritis etc. | +*2·6* | +*2·6* | +0·6 | +*2·3* | +*2·2* | −*4·5* |
| Tonsillitis etc. | +*3·4* | +0·7 | −0·6 | +0·9 | −1·6 | −0·4 |
| Miscellaneous | +0·2 | −0·1 | +1·2 | +*2·1* | +*2·6* | −*3·1* |
| Bronchitis etc. | +1·1 | +*2·4* | +0·2 | −0·9 | +*2·2* | −*2·7* |

* See Table VII footnote for significance levels.

TABLE XII.—*Pre-onset Illness Risks: Effects of Progressive Shortening of the Pre-Death Period* (t *values in Mantel–Haenszel Analysis*)

| Illnesses[1] | Progressive components: R/S | R/L | S/L |
|---|---|---|---|
| Bronchitis etc*** | −*4·3* | −*7·9* | −*2·5* |
| Tonsillitis etc*** | −*3·3* | −*6·4* | −*2·0* |
| Enteritis etc*** | −*2·1* | −*7·1* | −*4·5* |
| Whooping cough* | −*3·0* | −1·5 | +0·6 |
| Fractures and burns* | −*4·1* | −*6·4* | −1·5 |
| Rubella* | −1·0 | −1·7 | +0·8 |
| Measles | −0·6 | −*2·3* | −1·1 |
| Scarlet fever | −0·9 | −*2·4* | −0·4 |
| Minor infections | −*2·6* | −*12·1* | −*9·2* |
| Minor injuries | +0·2 | −*18·0* | −*17·5* |
| Miscellaneous | −0·5 | −*2·2* | −1·8 |
| *Chicken pox* | −*2·6* | −0·8 | +*2·3* |
| *Mumps* | −1·9 | −0·1 | +1·1 |
| *Allergies* | −*2·2* | −*2·4* | +0·5 |

[1] *** Significant chi-squares in all 3 analyses.
* Significant chi-squares in the R/S analysis only.
Italics: non-significant chi-squares in all 3 analyses.
— risk increasing with progressive shortening of the pre-death period.
+ risk increasing with progressive lengthening of the pre-death period.
(Significance levels as in footnote to Table VII.)

biased reporting of pre-onset illnesses by mothers of live and dead children; or some of the non-fatal illnesses having delayed (carcinogenic) effects; or some of the cancers having latent-period effects which had lowered resistance to other diseases. In practice, the different origins would be associated with different patterns of significance. For instance, any effects of biased reporting would not affect the R/S analysis; and in the other analyses minor illnesses in the shortest period would be more affected than other illnesses.

The second postulated effect would probably have different effects on RES neoplasms and solid tumours, and might involve viral infections more than other illnesses. Also, since cancers have long latent periods this effect should produce much higher *t* values for remote than for recent attacks.

Finally, the third postulated effect would certainly affect RES neoplasms more than solid tumours, infections more than other illnesses, secondary infections more than primary ones, and recent more than remote attacks. Therefore, there would be higher chi-squares for the 3 illness groups which included secondary infections than the other groups in the R/L than the S/L analysis. Also the progressive component $t$ values would be indicative of a mounting risk with progressive shortening of the pre-death period, and (most important) the R/S analysis would be affected as well as the R/L analysis.

### RESULTS

### *R/S analysis*

The 3 illness groups which included secondary infections had chi-squares ranging from 26·4 to 11·8 (Table VI). Only 3 of the remaining 11 groups had similar findings, and they were whooping cough (17·4) fractures and burns (17·3) and rubella (13·6). For the 3 groups representing secondary infections, the $t$ values for the shortest pre-death period ranged from +4·2 to +2·0 (Table VII) and the progressive component $t$ values from −4·3 to −2·1 (Table XII).

Therefore, in a fully controlled analysis of 2 groups of dead children (RES neoplasms and solid tumours) the null hypothesis was firmly rejected in favour of the theory which expects any cancer with direct involvement of the immune system to begin undermining resistance to infections before it is clinically recognizable.

### *R/L analysis*

There were exceptionally high chi-squares for the groups of minor injuries (369·7) and minor infections (161·6). Also, the chi-squares for 3 mixed groups of primary and secondary infections ranged from 66·6 to 53·8 (Table VIII). In addition there were significant chi-squares for fractures and burns (41·6), scarlet fever (13·0) and measles (11·7). For 5 illness groups with exceptionally high chi-squares (over 50·0), the $t$ values for the shortest pre-death period ranged from +11·1 to +6·2 (Table IX) and the progressive component $t$ values from −18·0 to −6·4 (Table XII).

Therefore, in a fully controlled analysis of a situation which allowed ample scope for biased reporting by mothers of live and dead children, there was definite evidence of such an effect. The evidence also suggests that mothers can easily recall a trivial complaint if it is shortly followed by a cancer death, but have difficulty in recalling similar complaints of healthy children.

### *S/L analysis*

The chi-square for minor injuries (367·9) was virtually the same as in the R/L analysis, but the values for 4 mixed groups of primary and secondary infections (including minor infections) though statistically significant, were much lower than the ones recorded in the R/L analysis (Table X); and only one of the other illness groups (miscellaneous) had a chi-square >11·1. For the group of minor injuries, and 3 of the infection groups the $t$ values for the shortest pre-death period ranged from +10·7 to +2·6 (Table XI) and the progressive component $t$ values from −17·5 to −2·5 (Table XII).

Taken in conjunction with the R/L analysis, these findings are suggestive of (i) under-reporting of minor injuries and infections by mothers of live children, (ii) equally good reporting of highly infectious diseases by the mothers of live and dead children and (iii) greater differences between RES neoplasms and live controls

TABLE XIII.—*Illness Risks for RES Neoplasms in 5 Pre-death Periods Relative to Solid Tumours (R/S) and Live Controls (R/L)*

| Pre-death periods | | Bronchitis etc. R/S | Bronchitis etc. (R/L) | Tonsillitis etc. R/S | Tonsillitis etc. (R/L) | Enteritis etc. R/S | Enteritis etc. (R/L) |
|---|---|---|---|---|---|---|---|
| Under 12 months | (0) | 2·1 | (2·5) | 1·6 | (2·6) | 1·5 | (3·0) |
| 12–23 months | (1) | 1·1 | (1·6) | 1·3 | (1·4) | 1·2 | (2·2) |
| 24–35 months | (2) | 1·4 | (1·5) | 1·0 | (1·1) | 1·5 | (1·5) |
| 36–47 months | (3) | 1·5 | (1·5) | 1·4 | (1·6) | 0·6 | (0·9) |
| Over 4 years | (4) | 1·0 | (1·3) | 1·2 | (1·1) | 1·0 | (1·3) |

than between solid tumours and live controls in relation to recent attacks of bronchitis, tonsillitis etc.

*Illness risks from RES neoplasms relative to solid tumours and live controls*

The results of each Mantel–Haenszel analysis included relative-risk estimates for each test-factor level, some of which are shown in Table XIII. For remote attacks of bronchitis, tonsillitis and gastroenteritis, there were negligible differences between the test group (RES neoplasms) and 2 control groups (cancer controls and live controls) but for recent attacks (*i.e.* within a year of the fatalities) the risk was 50–100% higher for the test group than for the cancer controls, and 250–300% higher for the test group than for the live controls.

### DISCUSSION

According to a fully controlled analysis of data from an ongoing retrospective survey of childhood cancers, there is probably a period of several months before the clinical onset of certain childhood cancers when infection risks are abnormally high. This is probably due to cancers of the immune system having latent-period effects which either prevent normal development of the affected cell systems (exceptionally young cases) or reduce the level of immunological competence (older cases).

It was only possible to compare the illness risks of children who later developed RES neoplasms (test group) with children who either developed solid tumours (cancer controls) or were representative of normal children (live controls). These comparisons showed that in relation to several primary infections (which were also highly infectious diseases, *e.g.* measles) there was little or nothing to choose between the 3 groups of children. However, in relation to 3 mixed groups of primary and secondary infections (which are only common among infants and substandard children) there were significant differences between the

TABLE XIV.—*Estimated Effects of Two Components of the Pneumonia Risks of British Children Born* 1940–1950. *Including Estimates of the Special Effects Associated with Preleukaemia**

* Sources: Kneale (1971), OSCC data, and official statistics of mortality.

test group and both control groups, differences which increased with progressive shortening of the pre-death period.

Although the data were only suitable for detecting the non-lethal component of any infection risks associated with precancers, an earlier study, based partly on the Oxford Survey and partly on official statistics of mortality, demonstrated a lethal component of the risk, and showed that this was responsible for more preleukaemia deaths before antibiotics were discovered than in recent years (Kneale, 1971). By combining the 2 studies, we have produced estimates of immediate and delayed effects of both components, and made them applicable to British children born between 1940 and 1950 (Table XIV).

According to these estimates, unrecognized leukaemias or latent-period deaths ascribed to pneumonia were twice as common as recognized cases, and for every child who had recovered from an attack of pneumonia before developing leukaemia there were 7 or 8 children who died from pneumonia during the terminal phase of preleukaemia.

In the light of these observations, it is hardly surprising that the discovery of antibiotics was followed by an upsurge of acute leukaemia deaths, followed by a more gradual rise in the death rates for other RES neoplasms. The extra cancer deaths were first concentrated in countries with high standards of medical care and in infection-sensitive age groups (Hewitt, 1955) but eventually all countries experienced similar changes, which gradually brought the age distributions of their leukaemia and lymphoma deaths into line with the ones which have been recorded in this country since 1968 (Stewart, 1972).

A single exception to the general rule exists, inasmuch as there is no country which has recorded a significant increase in the number of myeloid-leukaemia deaths within 5 years of birth. However, this can be explained by assuming that, when cancers combine near-conception origins with rapid growth rates and involvement of the immune system, they may end by making a newborn child totally dependent upon passive immunity for survival. Consequently, latent-period deaths might take the form of cot deaths rather than infection deaths (Stewart, 1975, 1977).

Finally, according to the estimates in Table XIV an attack of pneumonia which coincides with an advanced stage of preleukaemia is at least 50 times as likely to prove fatal as a "normal" attack. Consequently, the OSCC-based estimates of the cancer risks associated with foetal irradiation which were published in 1970 (Stewart and Kneale) are only directly applicable to children with similar infection risks.

This conclusion has important consequences for survivors from the 1945 atomic explosions, since an inevitable consequence of any nuclear holocaust is an exceptionally high infection-death rate for several years after the event. These early deaths are bound to have a disproportionate effect on infection-sensitive individuals. Therefore, it is clearly dangerous to use a population of A-bomb survivors to study the precise nature of the relationship between ionizing radiation and human cancers.

An example is a 10-year follow-up of 1250 A-bomb survivors (who were exposed *in utero* to less than 500 rad) which only succeeded in identifying one (non-leukaemic) cancer death. According to OSCC estimates of risk the expected number of radiogenic cancers was 20·0, and according to national statistics the expected number of non-radiogenic cancers was 0·82 (Jablon and Kato, 1970). The wide discrepancy between the first 2 numbers has often been quoted as a reason for doubting the validity of OSCC estimates. However, unless human foetuses are totally insensitive to the leukaemogenic effects of radiation, there should have been some cases of leukaemia among the A-bomb survivors. Therefore, we can only suppose that the proportion of unrecognized leukaemias (due to latent-period abortions or deaths) was much higher in this population than in the one covered by the Oxford Survey.

The Oxford Survey data were collected by a nation-wide network of doctors attached to County and County Borough Health Departments. The costs of the Mantel–Haenszel analysis were covered by the United States Department of Health, Education and Welfare (Contract Number 223-76-6026 negotiated by the Bureau of Radiological Health).

### REFERENCES

HEWITT, D. (1955) Some Features of Leukaemia Mortality. *Brit. J. prev. soc. Med.*, **9**, 81.

JABLON, S. & KATO, H. (1970) Childhood Cancer in Relation to Prenatal Exposure to Atomic Bomb Radiation. *Lancet*, ii, 1000.

KNEALE, G. M. (1971) The Excess Sensivity of Pre-Leukaemics to Pneumonia. A Model Situation for Studying the Interaction of an Infectious Disease with Cancer. *Brit. J. prev. soc. Med.*, **25**, 152.

KNEALE, G. W. & STEWART, A. M. (1976*a*) Mantel–Haenszel Analysis of Oxford Data. 1. Independent Effects of Several Birth Factors Including Fetal Irradiation. *J. natn. Cancer Inst.*, **56**, 879.

KNEALE, G. W. & STEWART, A. M. (1976*b*) Mantel–Haenszel Analysis of Oxford Data. II. Independent Effects of Fetal Irradiation Subfactors. *J. natn. Cancer Inst.*, **57**, 1009.

KNEALE, G. W. & STEWART, A. M. (1977) Age Variation in the Cancer Risks from a Foetal Irradiation. *Br. J. Cancer*, **36**, 501.

MANTEL, N. (1963) Chi-square Tests with One Degree of Freedom: Extensions of the Mantel–Haenszel Procedure. *J. Am. statist. Ass.*, **58**, 690.

MANTEL, N. & HAENSZEL, W. (1959) Statistical Aspects of the Analysis of Data from Retrospective Studies of Disease. *J. natn. Cancer Inst.*, **22**, 719.

STEWART, A. M. (1972) Epidemiology of Acute (and Chronic) Leukaemias. In *Clinics in Haematology*, Ed. S. Roath. London: W. B. Saunders, p. 3.

STEWART, A. M. (1975) Infant Leukaemias and Cot Deaths. *Br. med. J.*, ii, 605.

STEWART, A. M. (1977) Factors Affecting the Recognition of Childhood Cancers: Respiratory Infections, Cot Deaths and Season of Birth. *Pediat. Dig.*, **19**, 9.

STEWART, A. M. & BARBER, R. (1962) Survey of Childhood Malignancies: Progress Report. *Med Offr*, **107**, 3; and *U.S. Publ. Hlth.*, **77**, 129.

STEWART, A. M. & KNEALE, G. W. (1970) Radiation Dose Effects in Relation to Obstetric X-rays and Childhood Cancers. *Lancet*, i, 1185.

WORLD HEALTH ORGANIZATION. Manual of the International Statistical Classification of Diseases, Injuries and Causes of Death (8th Revision). Geneva, 1967.